# Growth Quakes and Stasis Using Iterations of Inflating Complex Random Matrices

**DOI:** 10.3390/e25111507

**Published:** 2023-10-31

**Authors:** Henri Benisty

**Affiliations:** 1 Laboratoire Charles Fabry, IOGS, Université Paris-Saclay, 2 Av. Fresnel, 91120 Palaiseau, France; henri.benisty@institutoptique.fr; Tel.: +33-1-6453-3286; 2 LIED Laboratory, Université Paris Cité, 5 rue Marie-Andrée Lagroua Weill-Hallé, 75205 Paris, France

**Keywords:** random matrices, growth, economy, ecology, evolution, ergodicity, inflation

## Abstract

I extend to the case of complex matrices, rather than the case of real matrices as in a prior study, a method of iterating the operation of an “inflating random matrix” onto a state vector to describe complex growing systems. I show that the process also describes in this complex case a punctuated growth with quakes and stasis. I assess that under one such inflation step, the vector will shift to a really different one (quakes) only if the inflated matrix has sufficiently dominant new eigenvectors. The vector shall prefer stasis (a similar vector) otherwise, similar to the real-valued matrices discussed in a prior study. Specifically, in order to extend the model relevance, I assess that under various update schemes of the system’s representative vector, the bimodal distribution of the changes of the dominant eigenvalue remains the core concept. Overall, I contend that the punctuations may appropriately address the issue of growth in systems combining a large weight of history and some sudden quake occurrences, such as economic systems or ecological systems, with the advantage that unpaired complex eigenvalues provide more degrees of freedom to suit real systems. Furthermore, random matrices could be the right meeting point for exerting thermodynamic analogies in a reasonably agnostic manner in such rich contexts, taking into account the profusion of items (individuals, species, goods, etc.) and their networked, tangled interactions 50+ years after their seminal use in R.M. May’s famous “interaction induced instability” paradigm. Finally, I suggest that non-ergodic tools could be further applied for tracking the specifics of large-scale evolution paths and for checking the model’s relevance to the domains mentioned above.

## 1. Introduction

Thermodynamics has the virtue of embedding a myriad of degrees of freedom into a simple collective response to system boundary conditions, based on maximum likelihood for equilibrium states. For very global systems, such as the economy or the planet’s ecological system, however, which are fed by external sources such as the sun, the challenge is different since non-equilibrium systems must be described, while the variety of their constituents is huge. The idea of a “simple collective response” thus becomes unlikely. Rather, a description of the largest events becomes the logical focus, hence a study of the role of stability and instability. It is now 50+ years since R.M. May [1,2] introduced the use of random matrices to study the stability of ecological systems, pointing out that the largest eigenvalues related to the system stability were an interesting “proxy” to tackle stability issues while ignoring much of the system’s detail (hence, a thermodynamic inspiration).

Nowadays, while May’s approach is still debated (i.e., whether more interactions beget instability in real ecological systems), the description of our own economic growth as actors of the Anthropocene relies on extreme simplifications. These are, for instance, the GDP (gross domestic product) or sectorial analysis limited to a few dozen sectors. Big data, on the other hand, are intensively exploited in the financial realm. Some prominent contributors (J.-P. Bouchaud [3] and M. Smerlak [4], for instance, among others) are exploring uncharted interdisciplinary territories with all the power of statistical physics and random matrices theory (RMT). 

Almost all of the literature on RMT, however, studies matrices of a given size N and the asymptotics N→∞. For instance, while it is well known that for real matrices $M\in \;R^{N}\times R^{N}$ (symmetric or from the Ginibre set), the largest eigenvalue $\lambda_{max}$ is distributed in an interval whose width scales with $N^{-2/3}$, given by the Tracy–Widom law (see [5] for a good analytical approximation). But how the largest eigenvalues and eigenvectors evolve when adding an extra row and column (N→N+1) is hardly documented (with a provision for the so-called “cavity method” [6] used for the recursive deduction of matrix properties, in the case of N+1→N).

In a recent contribution [7], the “matrix inflation” proposal was put forward to describe the growth of complex systems with a profusion of distinct “elements”. It was most notably aimed at describing their “punctuated” evolution according to the paradigm proposed by S.J. Gould for biological evolution: the occurrence of long stasis separated by short “quakes” [8,9]. The economy is another domain marked by a long series of technical disruptions. These disruptions were accelerated by the introduction of fossil energies two centuries ago, with such fuels becoming a conundrum for the future of societies. The core idea of the “matrix inflation” proposal is that the time-wise addition of a new row and column to an “iteration matrix” gives the representative vector a kind of “kick” that is adapted to model the role of successive innovations. The variable role of innovations, kicking large changes or not, is also a tenet of ecological systems’ evolution. The adoption of a new paradigm (for either case) occurs when the dominant eigenvalue and eigenvector make a leap instead of incrementally evolving under this action. This is possible in a privileged manner in a non-Hermitian setting because the moduli of extreme eigenvalues can leapfrog each other. In a Hermitian setting, on the contrary, the repulsion of neighbor eigenvalues translates into a no-leapfrog rule and an anti-crossing eigenvector exchange scenario (although, if not in an adiabatic limit, one could invoke Landau–Zener tunneling, for which a study within a non-Hermitian setting has recently appeared [10]). 

The issue of what is a “quake” in the economy is not obvious, because some global quantities still show some continuity in their trends. One example is the correlation of PEC (primary energy consumption) to GDP. It holds well on the 1820–2020 interval [11] (linear until 1920, sublinear from 1920 on), in spite of major events (WWI and WWII, flu epidemics, collectivized economy in the USSR, and the Cold War) and major overhauls in energy choices. Wood, coal, and oil have been added to the mix, which is still made from mostly fossil-sourced and carbon-spewing energies. Some minor quakes are apparent [11]; however, it is appropriate to emphasize that technical innovation has no privileged time scale: while, e.g., in France, cars caused the horse market to fully collapse within a decade pre-WWI, nuclear power plants and ammonia synthesis to mass-produce fertilizers were ramped up over three decades in the second half of the 20th century. Thus, it is likely that each technological disruption has had only a weak individual effect on our thermodynamic fate. To capture the scale of the changes to be made in our material civilization, and compare it to known “quakes”, a model of our growth and of its internal dynamics in terms of the profusion of objects and of the network of interaction would be enlightening. This was, remotely, the scope of the previous contribution [7]. 

In this paper, I re-examine the operation of this model with the aim of establishing more thoroughly to what degree the succession of dominant eigenvalues/eigenvectors is its principle of operation. It is, thus, an exploration of a less-explored swath of random matrix theory, with the aim of consolidating its further use to describe large real systems. The emerging features could also trigger new explorations of our way to cast the complexity of our society’s path in innovation. This may be useful to tame the trends of energy use that have been emerging, for which current mitigation strategies have unclear perspectives. 

Furthermore, I examine the case of entirely complex matrices, in which eigenvalues do not come in conjugate pairs, so that some tracking tools like the Rayleigh quotient have a simpler use. The methodology is, thus, to assess the role of a renewal strategy when “inflating” and “iterating” the model’s matrices by tracking the eigenvalues and the Rayleigh quotient. The latter is a good tool to capture the way an eigenvector evolves, with quakes and stasis, or with a more continuous evolution. One point of this methodology is to relate a discrete model of matrices, whereby a change in size is the key event within the more continuous frame of real-world evolution. Thus, I explore how the model works depending on the way it is iterated in time.

Once such a basis is consolidated, the use of the model to deal with the profusion of actual objects/goods in economy (or of species/individuals in ecosystems) could be developed. A possible perspective, in the spirit of thermodynamics, would be to apply various metrics of non-ergodicity (the subject has been interestingly linked to inequalities in the economy by O. Peters et al. [12,13,14,15]). This approach is complementary to the “microscopic” aspect of the renewal strategy. The issue of non-ergodicity of the evolution described by the model is certainly an important “macroscopic” aspect. It can be approached from the angle of the type of long-term memory the system possesses of its past, of the way in which its current trajectory is dictated by its previous states. I will propose an initial exploration of this question.

The knowledge gained through these explorations could then be integrated into a more general vision. A desirable objective is to find the proper scaling in terms of the efforts needed to modify growth in a way compatible with current IPCC reports, for example: how big are the “quakes” the economy needs to shift to an acceptable trajectory. If we refer to related scientific areas, in order to act in a highly entangled economy, the desired new “quakes” and the following “stasis” could be better defined in the grammar of network theory, which closely parallels that of non-Hermitian Hamiltonians and RMT. Physicists practicing non-equilibrium thermodynamics would thus have the opportunity to contribute to radical transformations. Econophysics has shown some aspects of this understanding, but, in my opinion, it has failed to describe the “network complexity” of the real world. In a related area, impressive studies of actual networks have provided many new insights (e.g., labor flows and firm size in the work of R. Axtell [16]), but with more emphasis on the nature of the links between entities (firms, people) than in relation to goods and energy. Despite the substantial changes that have occurred in the capitalist era (from the single-earner family of the industrial era to the two-earner family of the post-industrial era for example), changes captured through such a prism might constitute only a partial picture of our material society, limiting its ability to foster large-scale changes.

After defining these aims and examining the related methodological issues, the paper proceeds as follows: In Section 2, I recall the main basis of the model, and the quantities I have tracked to give a reasonable idea of the relevance of the central concept of “dominance of successive eigenvalues”, which I see as an asset in meeting the knowledge challenges suggested above. In Section 3, I present the behavior of the model under different “iteration schemes” and show that only a subset of the eigenvalues is concerned if slower, “sluggish” iteration schemes are implemented. I show that there are only minor differences between the Ginibre set of (purely real) random matrices and the (fully complex) complex set with respect to the eigenvalue changes upon N→N+1  “inflation”. A “pragmatic” sensitivity analysis concludes this section. I discuss the possible role of non-ergodicity in Section 4 through some perspectives, and I conclude in Section 5.

## 2. The Matrix Inflation Model and Its Typical Output

The two aspects I have combined in the matrix inflation model [7] can be described as follows. 

(A) First, in an iteration scheme $U\left(t+1\right)=MU\left(t\right)$ where M is an eventually large N×N matrix and $U\left(t\right)$ a discrete-time vector function ($t\in N^{*}$, akin to those producing Krylov suites), the vector $U\left(t\right)$ tends toward the eigenvector with the largest modulus eigenvalue $\lambda_{max}$ (in short the DEV(N), dominant eigenvector at size N). Possible degeneracies appear for real nonsymmetric M ($M_{jk}\in R$) with a majority of complex eigenvalues coming as conjugate pairs, but only accidentally for complex eigenvalues ($M_{jk}\in C$). Moreover, for random matrices with all elements taken as the scaled centered normal law, $M_{jk}\in N\left(0,1\right){\left(N\right)}^{-1/2}\;$ for the real case and $M_{jk}\in [N\left(0,1\right){+iN\left(0,1\right)]\;\left(2N\right)}^{-1/2}$ for the complex case, a majority of eigenvalues cluster asymptotically in the unit-radius disc. A small fraction remains beyond it, comprising the extreme ones, lying in the “tail” of the distribution according e.g., to various versions of the Tracy–Widom laws [5,17]. Note that this tail is substantially more extended towards the very largest outliers in the complex case than in the real case.

(B) Secondly, when the size of the matrix is increased N→N+1 (one new row and one new column, the rest unchanged), the new eigenvectors are generally far from orthogonal to the previous ones. Thus, the DEV(N) has a sufficiently large projection on DEV(N+1) acting as a large seed and resulting in making the subsequent iterations of M at size N+1 converge to DEV(N+1). The salt of the process is that the frequent case is a limited change in DEV(N+1) relative to DEV(N), regardless of the exact change in $\lambda_{max}(N+1)$ relative to $\lambda_{max}(N)$, whereas the rare case is that a widely different DEV(N+1) comes out: this rare case is logically associated (in the sense of “closer to” using an appropriate scalar product) with one of the large eigenvalues that was just below $\lambda_{max}\left(N\right)$ before inflation, and that took advantage of the extra row and column, so to speak, to leapfrog above the eigenvalue of DEV(N). This rare event then corresponds to a quake. 

The mechanism that consists of making $U\left(t+1\right)=MU\left(t\right)$ enough times (say r repetitions) at a given size to attain a vector close to DEV(N), and then, at later times t+r, to inflate the matrix by one new row and one new column, naturally provides a sequence of quakes and stasis. And it also implies that the dominant eigenvectors across the quakes are seemingly unrelated. This is exactly the feature of “growth with disruption”, which I see as a welcome, if highly stylized, representation of the actual Schumpeterian growth picture in economics known as destructive creation (when an innovation entirely displaces a previous dynamic equilibrium) [18,19]; or a representation of species evolution with punctuated equilibria à la Gould [8,9].

At this point, a few simple remarks can be made. Convergence when using a matrix of a given size,

(1) $$U\left(t+r\right)=M^{r}U\left(t\right),$$

depends on the difference between the dominant eigenvalue and the next (degeneracy allowing). The scaling of the convergence is exp⁡(−|Δλ|r). The starting point depends how a previously converged vector $U\left(t\right)$, which became an eigenvector pre-inflation (a right eigenvector, in my formalism), projects onto the dominant (still right) eigenvector post-inflation. With my normalized matrices, |Δλ| could be considered to scale like $N^{-1}$. But the inflation process itself and the fact that the system is considered just after a “leapfrog” of the previously second dominant eigenvector (I justified this assertion with an auxiliary drift-diffusion model leading to the same kind of *q*-exponential law of quakes spacing as the law of spacing observed in the actual model [7]) could lead to a smaller-than-normal spacing. Nevertheless, I observe that products reaching r/N≳0.3 already lead to bona fide model convergence for the ≲10–15  quakes observed at the values N∼ a few hundred. As for the projection, it is delicate to guess its scaling, as it demands careful tracking of the eigenvectors through the inflation stage to define it correctly. Indeed, this is typical of the subtleties of matrix inflation analysis.

**Figure 1:**
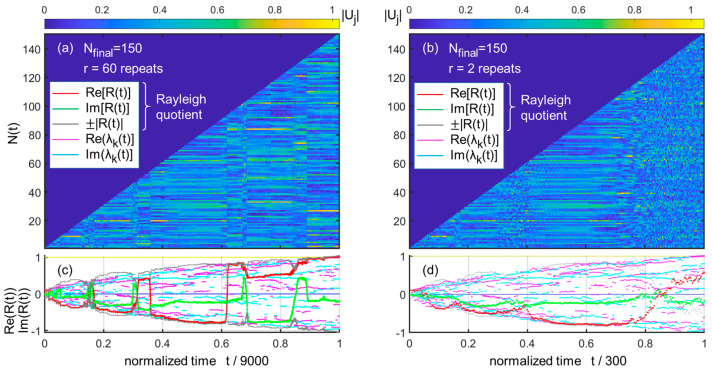
Role of growth rate for $N_{final}=150\;\;$ (**a**) eigenvectors vs. normalized time t for r=60 repeats (thus, $t_{final}=150\times 60=9000$); the stasis with the different dominant eigenvectors are clearly visible, e.g., from N=55 to N=90 (t/9000≈0.37 to t≈0.60); (**b**) eigenvectors vs. normalized time t for r=2 repeats (thus, $t_{final}=150\times 2=300$); the shorter stasis is “missed” by evolution. The snowy patterns (e.g., at abscissa > 0.8) are typical of oscillations. (**c**) Plot of Rayleigh quotient (Re and Im in colors, modulus in grey) and the real and imaginary parts of the few largest eigenvalues of the local matrix of size N (see legend for colors) for r=60 repeats; (**d**) same for r=2 repeats, note the smeared transitions of the real and imaginary parts of the Rayleigh quotient.

I can now present the results of Figure 1a–d, whereby I use parameters such that the convergence is good in case (a) with $r/N_{final}=0.4$ and weak in case (b) with only $r=2\;(r/N_{final}=0.0133)$. Here, $N_{final}$ is the final size of the matrix in the computation (done with Matlab^©^). I discard the absolute growth of the eigenvector at this stage, as it is easily rescaled arbitrarily. The Figure 1a,c thus essentially show, in the form of two color maps, the growth of the vector $U\left(t\right)$ with its stasis and quakes, through the moduli of its (nonzero) components. The final time $t_{final}=N_{final}r$ is normalized to 1 for simplicity. Below the figure, to find out whether the “local dominant eigenvalue” is adopted (if so, one can reasonably infer that the corresponding eigenvector is also adopted, although the non-Hermitian setting calls for certain provisions), I make use of the simple tool of the Rayleigh quotient [20] R(t)∈C:

(2) $$R(t)=\frac{\langle U(t)\left|M^{\left(N(t)\right)}\right|U(t)\rangle }{\langle U(t)|U(t)\rangle },$$

where $M^{\left(N(t)\right)}$ indicates that I restrict the operation to the active part ($N\left(t\right)\times N(t)$) of the matrix (technically, I first draw a $N_{final}\times N_{final}$ random matrix, and use its subblock $N\left(t\right)\times N(t)\;$ at the appropriate time step).

Below the two color maps, I plot the corresponding real and imaginary parts of the Rayleigh quotient $Re\left(R\left(t\right)\right)$ and $Im\left(R\left(t\right)\right)$, as well as those of the 6 largest eigenvalues $Re\left(\lambda_{k}\left(t\right)\right)$ and $Im\left(\lambda_{k}\left(t\right)\right)$ in Figure 1c,d.

Comparing the two cases, it is clear that for Figure 1c,d, r=2 is largely insufficient to converge to the local eigenvalue. The pattern of eigenvalues themselves features either a smooth variation upon an inflation step, or in some case a more severe disruption. Just as in Francis Galton’s old (about as old as Darwin’s) polyhedron explanation for irreversible abrupt changes in evolution (see the figure, e.g., in Erwin’s review of Gould’s work [21]), there are intrinsic tipping points in the inflation process, at least as far as the ranked eigenvalues result is concerned. In the case of r=2, it can be seen that the convergence to the eigenvalues is prevented by the ongoing inflation steps. In this case, the tipping effects are smoothed, but the dynamics may still exhibit abrupt sequences.

I am now in position to examine the various results of the next section.

## 3. Results on the Operation of the Matrix Inflation Model 

### 3.1. Inflation Model and System Inertia

Let us first study the time iteration in relation to “inertia”. If a vector is assumed to describe a real system (economic or ecological), then this system has some inertia. It would not shift from a matrix to an inflated matrix at once, as it takes time to have an innovation adopted. One could think of innovation diffusion, i.e., the ability of other innovations (other inflation steps) following a given innovation to incorporate it and amplify its effects. This would wash out the disruption effect of any given innovation, giving rise to “variable dynamics” all along time, and making it difficult to directly track anything “Schumpeterian” [18,19]. This approach cannot be ruled out, but it reduces the tipping (eigenvalue leapfrogging) behavior of the vector to a witness to the adoption of “all the rest before” in a rather indistinct way. 

Another way of introducing inertia into the model is to “mix” the output MU(t) and the previous vector $U\left(t\right)$ itself to obtain U(t+1), with a simple linear mixture governed by a coefficient α:

(3) $$U\left(t+1\right)=(1-\alpha )U\left(t\right)+\alpha MU(t),$$

While the idea is fairly intuitive (the vector is asked to travel only a certain path on the way from $U\left(t\right)$ to $MU\left(t\right)$, the arrival point), the operation is not neutral, not as neutral as a mere delay would be. It amounts to replacing M by

(4) $$M_{\alpha }=\left(1-\alpha \right)I+\alpha M,$$

whose *eigenvectors* are obviously the same, but whose *eigenvalues* are

(5) $$\lambda_{\alpha ,k}=\left(1-\alpha \right)+\alpha \lambda_{k},$$

For these modified eigenvalues $\lambda_{\alpha ,k}$, the leading pattern in complex space is not the unity disc ($\left|\lambda_{k}\right|\leq 1)$ but a disc smaller by a factor α, centered at $c_{\alpha }=1-\alpha$ (on the positive real half-axis if α is real). Then, there is no reason for the largest-modulus eigenvalue to be the same, unless it lies along the $c_{\alpha }$ line, say the real axis if $Im\left(c_{\alpha }\right)=0$, or close enough depending on the competing eigenvalues of M. 

**Figure 2:**
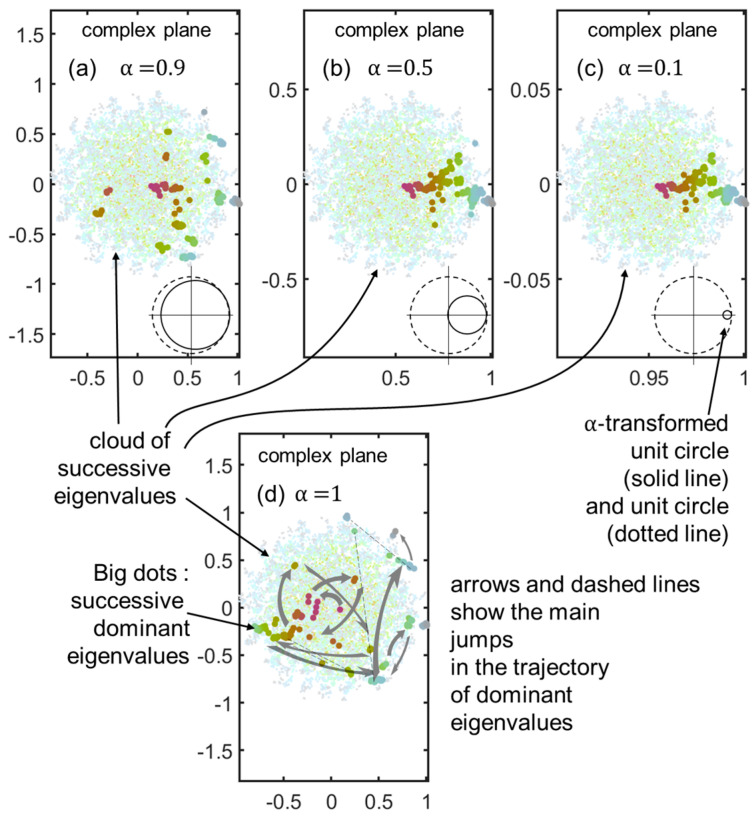
Fate of dominant eigenvalue as a function of renewal rate  α, associated with variable inertia. The eigenvalues of successive inflating matrices M or the renewal-modified version $M_{\alpha }$ are shown as small and light dots, with a sequence of hues going from brownish to greenish to blueish. Panels (**a**–**c**) show the values of α=0.1, α=0.5, and α=0.9, respectively, while panel (**d**) shows the original M. The inset shows the unit disc as shrunk and moved to be tangent to the left of z=1 by the transformation of Equation (5). The dominant eigenvalues are represented by the large darker dots, with a sequence of hues corresponding to the small and light dots (saturation enhancement, brown to green to blue-grey sequence). In the reference case (**d**), the dominant eigenvalue trajectory (grey arrows), while presenting the clusters of the quakes and stasis pattern, samples the unit disc rather randomly. For the other cases, the smaller the value of α, the more the dominant eigenvalue/(eigenvector) dots crowd to the right side close to z=1, and they therefore sample a different set from the reference set in (**d**).

A sampling study of the largest eigenvalue position for successive matrices M (of size $N\leq N_{max}=150$) for three representative values of , α ( α=0.1, α=0.5, and α=0.9) is shown in Figure 2a,c, along with the reference case ($M_{\alpha }=M,\;\alpha =1$) in Figure 2d. It is seen that as α diminishes, the set of successive largest eigenvalues tends to cluster on the real axis, while having just the same configuration as that of the reference case but reduced by the factor α.

**Figure 3:**
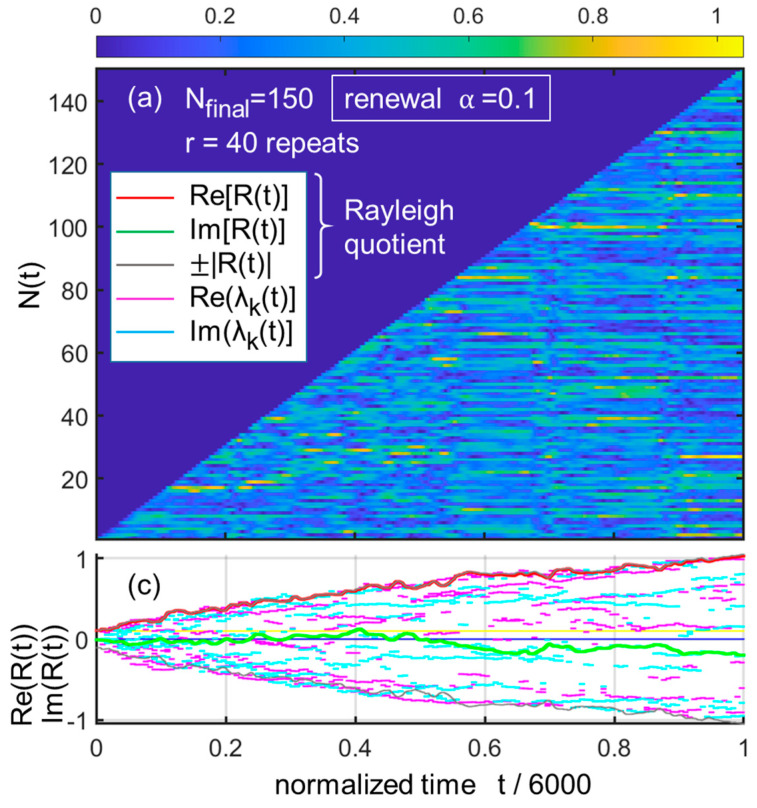
Same principle as Figure 1a,c but with a small renewal rate, α=0.1 and nearly as large a number of repeats (r=40). Note the different trajectory of the Rayleigh quotient, which remains real. Nevertheless, the appearance of stasis is still clear (the blurred aspect for low values of N is rather an oscillation regime).

Consequently, if the model is run with this prescription in the more “inertial” case α=0.1 and a still large number of repeats r=40, one sees in Figure 3 that the resulting vector is different (using the same random matrix draw as in Figure 1). One also sees that the Rayleigh quotient bounds its imaginary part $Im\left(R\left(t\right)\right)$ to a value well below unity, leaving the lion’s share to $Re\left(R\left(t\right)\right)$. 

At this point, the question arises as to how to induce inertia while preserving the same sequence of tipping point eigenvectors as in the original α=1 process. Formally, there is an angle φ∈[−π,π] such that, if $\alpha =ae^{i\varphi }$ with a>0, the rotation induced by the term $\alpha \lambda_{k}$ in Equation (5) brings the new largest eigenvalue to the outer edge of the rotated circle along the line $z={\rho e}^{i\varphi }$, hence ensuring that it remains the largest. But this involves adjusting the inertia according to the result, which weakens the degree of generality sought in such stylized models. But on the other hand, it provides an interesting feature as to how an innovation is “received” in a given society, economy, or ecosystem with its idiosyncratic inertia. The idea that innovations, more often than not, lead to a “dephasing” is a vocabulary used by early thinkers of modern technology such as Gilbert Simondon [22], suggesting that society goes first to adaptation stages and next to adoption stages of the innovation. This is of course a semantic game, but given the difficulty of finding models that include all systemic aspects of innovation-cum-profusion, the suggestion might attract some interest. One of the aspects of this difficulty of finding a model with a broad meaning with regard to these questions of innovation reception stems from the fact that we are in a world made up of very diverse societies that are nevertheless irrigated (if not constrained for many of them) by technologies developed by a few.

### 3.2. A Basic Comparison of Real and Complex Matrices

Next, I am looking for an elementary way to compare the core of the matrix inflation process when run with a real matrix and with a complex matrix. In the first case, if the initial vector $U\left(t=1\right)$ is real, then $U\left(t>1\right)$ remains real at all subsequent times. The Rayleigh quotient is therefore a purely real number. The fact that the eigenvalues are complex is compounded by the presence of conjugate pairs of eigenvalues/eigenvectors. The corresponding U has a structure analogous to a cosine with respect to the two complex exponentials (there is a decomposition of $U\left(t\right)$ as a sum of terms such as $\lambda_{k}U_{k}+\lambda_{k}^{*}U_{k}^{*}$, assuming ad hoc indexing by nonconjugate eigenvalues k). In practice, this leads to cosine-like oscillations in the elements of $M^{r}U(t)$, during the repeats. It is possible to use a modified matrix M that retains the same eigenvectors but forces the eigenvalues that cause oscillations (and which lie in a half plane) to be reduced to as small values as desired, effectively “killing” or at least “taming” these oscillations. It is also possible to eliminate the oscillatory process by signal processing, in order to obtain a clearer view of punctuated growth. These are partly artifactitious additions to the initial model, and are thus not the most appropriate approaches for retaining maximum generality. 

One of the interests of the complex case (M and U both fully complex) is to escape these specific unpleasant consequences of conjugate eigenvalues. The price to pay is the difficulty of establishing some correspondence with a real ecosystem or a real economy, described by real numbers. 

As for the core process itself, i.e., the way the eigenvalues can “leapfrog” each other (again, this view assumes that the eigenvectors can be tracked throughout an inflation step, which is generally possible, but not strictly always), it can be questioned how it is modified between the two cases. A simple way to probe this is to blindly examine the probability distribution function (pdf) of the quantity $\Delta_{N}=\left|\lambda_{max}^{\left(N+1\right)}\right|-\left|\lambda_{max}^{\left(N\right)}\right|$, the difference of the moduli of the “largest modulus eigenvalues” when going through the inflation step. As the matrices are not rescaled during the inflation (it was apparent from the $\sqrt{N}$ evolution of the eigenvalues in Figure 1, they are scaled to reach the unit radius at $N=N_{final}$), it is expected that the distribution is asymmetrical, favoring the tail on the long side ($\Delta_{N}>0$) over the small-side tail $\Delta_{N}<0$. Given the unit radius condition common to the real and complex cases, the coefficients have an appropriate factor of $\sqrt{2}$ in the real [7] vs. the complex case (discussed in this paper). 

**Figure 4:**
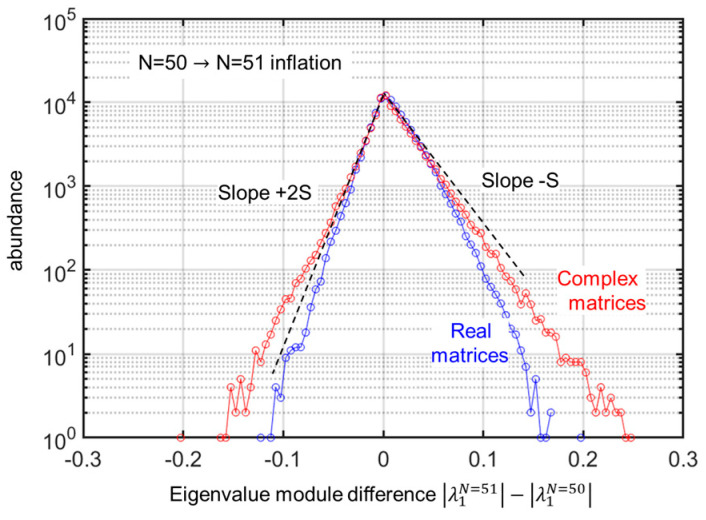
Comparison of the role of matrix inflation between real matrices and complex matrices. Using 100,000 draws of matrices of size 51×51  with the same semi-circle radius of 1, I compare the statistical distribution (pdf) of the change in eigenvalue modulus difference upon an inflation process $\left|\lambda_{1}^{N=51}\right|-\left|\lambda_{1}^{N=50}\right|$. The central part of the distribution (with abundance > 10% of max abundance) shows the same distribution for both types of matrices, with two different slopes using the logarithmic scale of abundance, differing by a factor of 2 (the smaller slope and thus the greater dispersion along the positive side of the abscissa since the difference $\left|\lambda_{1}^{N=51}\right|-\left|\lambda_{1}^{N=50}\right|$ is calculated along the increasing mean eigenvalue direction). However, for the extremes, the trend for complex matrices shows a “fatter” tail. These differences also correspond to the “fatter tail” of the largest eigenvalue at a given size.

Using enough draws, I obtain the pdf for both cases in Figure 4. They do not differ much, with similar but slightly different slopes on the y-log-scale purposely chosen for the graph, showing that there are two Poisson-type pdf on each side of the sharp maximum. This suggests that much of the model’s properties are dictated by the structural properties of the eigenvalue evolution. I mean here by structural the topology of the disk in the complex plane. Furthermore, the picture of a competition with drift and diffusion of the few largest eigenvalues is appropriate in both cases, real or complex coefficients. Furthermore, as the eigenvectors lie in the same (complex) space in both cases, the statistics relating to the evolution of the vectors throughout the quakes (correlation of components through the quakes) should also be similar, with tools such as scalar products offering similar analysis opportunities.

### 3.3. A Basic Sensitivity Analysis

**Figure 5:**
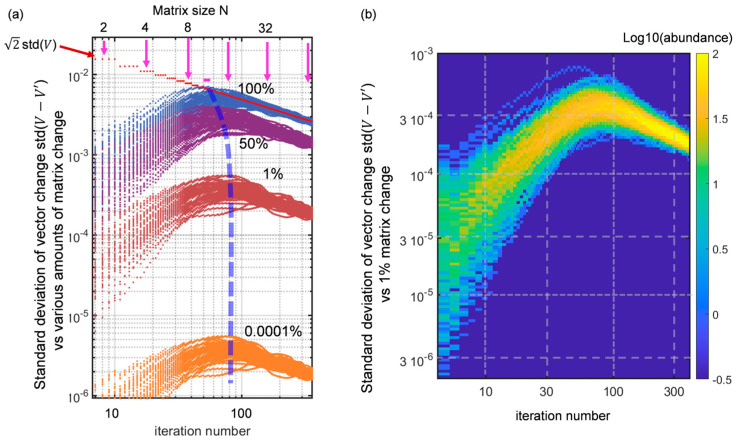
(**a**) Standard deviation of the $U\left(t\right)$ vector, $SD\left(t\right)=std\left(U\left(t\right)-U\prime (t)\right)$ for variably modified matrices $M^{\prime }={aM}^{\left(1\right)}+bM^{\left(2\right)}\;$, as a function of the number of iterations in log-log scales. The coefficient $b^{2}$ (in percent) is used to index the four curves shown, based on 50 draws. The top red curve is the natural upper limit based on the vector’s own standard deviation; hence $\sqrt{2}\;std\left(U\left(t\right)\right)$ for identically, normally-distributed $U\left(t\right)$ and $U\prime \left(t\right)$ with both having complex coefficients. The thick blue dashed line is a visual guide to the shifting maximum of the curves; (**b**) Color-coded histogram (log10(abundance), cf. color bar) for the same quantity $SD\left(t\right)$ and the case $b^{2}\;$= 0.01, based on a few thousand draws, giving a better view of the tails of the distribution at different points.

The model that I propose has two parameters, the number of repeats r and the renewal rate α. Their role has been assessed and the main limitations of the sensitivity analysis for these parameters can be guessed from the above results. As for the initial vector and the normalization, they are of no importance for the sequence of eigenvalues sampled during the inflation steps. The statistical choice of the random draws of the matrix is not a parameter in the usual sense, but it is interesting to ask what role it plays in the result. In other words, how sensitive is the resulting vector sequence when a matrix is modified? This is the “pragmatic” aspect of the ergodicity question that I shall examine in Section 5, paying more attention to the specific paths taken by vectors. A simple way of answering this question is to examine the extent of the standard deviation of the (complex) vector change when the matrix M is modifed. We thus track the real quantity $SD\left(t\right)=std\left(U\left(t\right)-U\prime \left(t\right)\right)$ along the iteration path, with $U\left(t\right)$ obtained from a given matrix $M^{\left(1\right)}$, and $U\prime \left(t\right)$ obtained from a modified matrix $M^{\prime }={aM}^{\left(1\right)}+bM^{\left(2\right)}$. The “mixing coefficients” obey $a^{2}+b^{2}=1$ to preserve the statistical properties of $M^{\prime }$. Figure 5a shows the resulting plots, for the set of mixing coefficients $b^{2}\in \{10^{-6}\;10^{-2}\;0.5\;1\;\}$ in four different colours and in the loglog plot, zooming in on the interesting part of the data. Only 50 draws of $M^{\left(1\right)}$ (and $M^{\left(2\right)}$) were used to preserve the visible information. In this study, r=5, N=150, $t_{final}=750$. The dark red dotted line at the top is the statistical upper bound of these differences, $\sqrt{2}\;std\left(U\left(t\right)\right)$, which is, in general, a nearly perfect straight line on such a scale, with a slope simply dictated by normalization (hence the steps, of which only the first are noticeable on the top left). The main point emerges directly from this loglog setting: starting from a given vector, the sensitivity reaches a maximum at a value that appears to depend on $b^{2}$ only in the large $b^{2}$ limit, and to be independent in the small $b^{2}$ limit, with a mean deviation essentially scaling as $b^{2}$. The vector cannot widely differ, obviously enough for small $b^{2}$ where the imprint of ${aM}^{\left(1\right)}$ remains strong, but not so obviously for stronger mixtures. I propose that this has to do with the fact that the vectors $U\left(t\right)$ do not have a “wild” distribution (see Figure 1 and Figure 3). Hence, they all probe the matrix content in a similar “averaging” way, approaching an asymptotic deviation more or less rapidly. However the sample for this figure is modest (50 draws). To obtain more details of the tails of the distribution, a color-coded histogram of the $b^{2}=0.01$ case has been produced and is shown in Figure 5b with 2500 draws of $M^{\left(1\right)}$. It confirms that the distribution of standard deviation $SD\left(t\right)$, which is my “proxy” for an elementary sensitivity analysis, has modest tails. Thus, the picture of Figure 5a with a modest spread of outliers is a fairly faithful representation of an elementary sensitivity analysis. In Section 4, I will question the model’s dependence on its “history” in another, less “pragmatic” way, also more connected to ergodicity.

## 4. Discussion

I have examined the extension of the growth model based on “matrix inflation” that I had previously introduced for the sole case of real matrices (non-symmetric, the Ginibre set) in [7]. As could be anticipated from the basic idea of eigenvalues “leapfrogging” one another through an inflation step, while carrying their eigenvector only marginally modified with them, the resulting dynamics of the inflation process appear very similar. I did not carry out the further analysis leading to the *q*-exponential law that I had found in [7], but the results seem qualitatively quite analogous, from the point of view of the spacing of quakes and the kind of changes they induce through the successive dominant eigenvectors.

I did not take into account absolute growth, which may occur more freely here given that eigenvectors do not come in conjugate pairs (leading to “cosine” oscillations during iterations). However, it is much more difficult to imagine that the absolute growth can have a deep meaning in terms of the “punctuated equilibria”, if one thinks of the classical issues of ecological systems, whereby proliferation is generally tamed by the ecosystem as a whole. Weeds, for example, have an enormous growth potential if one counts the ability to grow more than 100 seeds from a single one per year. Human agriculture can therefore be conceived as a race against the weed in time and space to obtain plants that provide, relatively slowly, staple food rather than many new seeds. But it is also known from the same systems that weeds are constrained to a much lower effective growth rate, except in invasion sequences (often created by man). In the case of invasive sequences, the issues at stake are rather those of qualitative growth rather than merely quantitative growth. Plants that have some kinds of robust rhizome (bamboo) or worse, that have a rhizome robust and surviving to subdivision in small pieces (e.g., Japanese knotweed, which circulates when soil is removed during river, road, and house works and redeposited) lead to striking invasions, while brambles and nettles are tamed after a while, e.g., as the forests that host them evolve over the years.

Apart from these general considerations, I have shown that even a relatively simple change in the model, the “amount of renewing” of the new vector with the old one, has an important, subtle effect, in terms of eigenvalues. It will be interesting to see, looking at actual systems, what are the proper ways to understand their inertia, and whether it favors any of the eigenvalue patterns discussed above. I anticipate that forcing the eigenvalues to lie in a closer corner of the disc in the case of partial renewal (long inertia) would also lead to a different correlation pattern of successive eigenvectors, which will undoubtedly deserve further investigation.

A final consideration concerns non-ergodicity. The “pragmatic” sensitivity analysis in Figure 5 has given an estimate of the ensemble deviation. But it is clear that, as the model is run, one can say that each sequence (in fact, each random matrix M and, in practice, each $M_{final}$) is a particular case. The ensemble average of these sequences would clearly, asymptotically, wash out the details, and yield “gray”, “average” vectors containing no useful information. Furthermore, in the perfectly nonstationary context of growth, it is apparently impossible to define a sensible time-average to perform an ergodic test. This is a concern given that the model deals with quakes and crises, for the understanding of which non-ergodicity could play an important role. Such a role could be investigated in the apparently simple case of a geometric Brownian motion (GBM) series [12,13,14,15]. 

However, it is maybe possible to play the same game that historians are often asked to play, i.e., giving “some kind” of previsions (it takes more than rhetoric dispositions, here for fairness, to say that historians, as agents of a professional community, warn rather starkly that however elaborate their understanding of the past may be, it has no direct scientifically-based predicting power). Transposed into my framework, this means asking, for each given case, how much does the evolution in the past times (thus of concern for the few basic components), used as a temporal average, makes sense for future changes (which, at the time of prediction, may look like an ensemble average). Of course, this is admittedly a quite skewed vision of ergodicity, but the conversation on this point may receive more attention as more data are collected that carry quantities of “historic” information, which could at least be used to inform a variety of scenarios.

**Figure 6:**
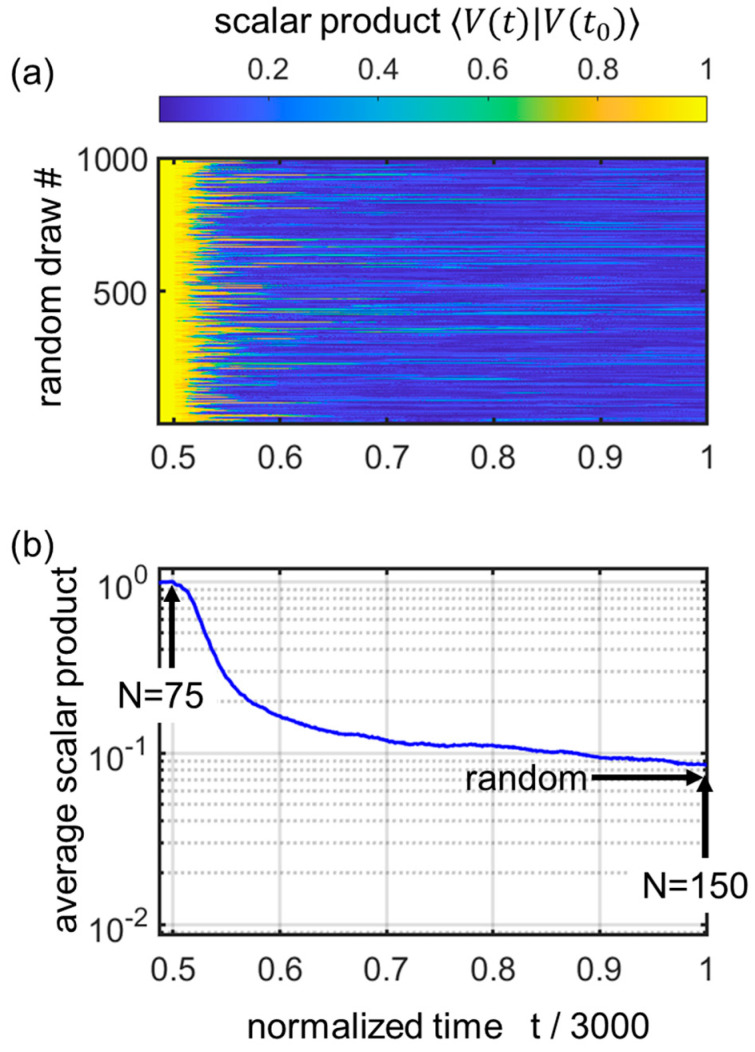
Memory study tackling the ergodicity issue. Vectors are iterated with different random realizations, after a given initial start and inflation from N=1 to N=75 (with r=20 repeats, thus up to $t_{0}=1500)$. Then, from N=76 to N=150, (t=1501 to t=3000), a set of 1000 random different inflation scenarios is considered. To see how the system forgets its past, I track the scalar products $\left|\langle V(t)|V\left(t_{0}\right)\rangle \right|$ on the “future” side of $t_{0}$. (**a**) Colormap of the 1000 scalar products moduli over time, showing variable “inheritance” scenarios. (**b**) Average of the 1000 cases. There is a rapid initial decay over a period typical of the *q*-exponential law of spacing (ΔN~10–15, Δt∼200–300, Δt/3000∼0.07–0.1). This is followed by a more sluggish decay towards the random limit, indicated by an arrow.

A first step in the proposed direction is shown in Figure 6. Here, the matrix is inflated only in one particular case up to “half-way” of the above simulation, hence N=75 (here with r=20, enough to qualitatively follow the DEV). Then, the rest of the inflation is performed with variable random additions of lines and columns while keeping the same “core” of the matrix (core size $N_{final}/2\times N_{final}/2$) which represents “the past”. By running different possible “futures”, one can see the role of “the past”, now in the ensemble manner as well as the time manner at will, thus somewhat addressing the tenets of ergodicity. More details can be grasped from the caption of Figure 6a,b. The chosen process reveals that memory, under these conditions chosen for preliminary exploration, clearly displays two time scales, a fast one related to the spacing of quakes (as described e.g., by a *q*-exponential law of [7]), and a longer time scale, during which a more “sluggish” decay to the mean value of the scalar product (∼0.072 for the case N=150) takes place. Thus, there are signs of long memory, with a possible appearance of non-ergodic aspects in the evolution of this model. I intend to address these in future work.

## 5. Conclusions and Perspectives

To conclude, I addressed some simple mathematical-physical issues related to the use of the matrix inflation model, bearing in mind its application to economic systems and ecological systems, in order to explain, among other things, their “quakes and stasis” patterns. I suggested that complex matrices, while unnatural for describing the usual quantities of economics, might be a tempting approach. The resulting evolution of eigenvectors obeys rules quite similar to those of a real matrix, and I defer to further work the full check that a q-exponential law also applies to the spacing of quakes in time, as in the real case [7]. And the avoidance of certain complexities that arise with conjugate pairs of eigenvectors could be considered a simplification.

I discussed how “inertia”, by partial renewal, was introducing an interesting bias on the set of successive dominant eigenvalues of the underlying matrix M, favoring those closer to the right of the disc (z→1) in the complex plane. The pattern of eigenvalue modulus evolution was also found to be very similar in appearance. There are now several ways to exploit the possible outcomes of such a domain. 

Some look challenging, such as importing the notion of ergodicity in a non-stationary context (although the network context could be useful to examine the issue, see [23] for an example of network evolution in a non-Hermitian context). Others are closer to what the existence of big data troves allows: exploring the growth dynamics as I have carried out earlier [7] for the Web Of Science (WOS) bibliographic records (via the number of journals in a few tested WOS domains), and tracking whether changes in the patterns map those among different eigenvectors of an inflating matrix, as I proposed here. Other possible domains for such attempts could be in catalogs that combine hierarchy and curation to a sufficiently high level. The European REACH legislation from the ECHA (European Chemical Agency) comes with a catalog of chemicals that could serve this kind of purpose. The ecosystem of semiconductor products could also lend itself to the exercise, as this specific industry maintains high supply standards to ensure its viability in the face of the rapid evolution of company in the sector. These proposed uses of non-Hermitian random matrices would then acquire one more universal characteristic feature, which adds to the many features of random matrices in general.

## Data Availability

My Matlab^©^ programs are available on reasonable demand.
